# Human Telomerase RNA: Telomerase Component or More?

**DOI:** 10.3390/biom10060873

**Published:** 2020-06-06

**Authors:** Maria Rubtsova, Olga Dontsova

**Affiliations:** 1 Chemistry Department and A.N. Belozersky Institute of Physico-Chemical Biology, Lomonosov Moscow State University, 119992 Moscow, Russia; 2 Center of Life Sciences, Skolkovo Institute of Science and Technology, Skolkovo, 143026 Moscow, Russia; 3 Shemyakin-Ovchinnikov Institute of Bioorganic Chemistry, Russian Academy of Sciences, 117997 Moscow, Russia

**Keywords:** telomerase, ribonucleoprotein particles, alternative function, telomere

## Abstract

Telomerase is a ribonucleoprotein complex that maintains the lengths of telomeres. Most studies of telomerase function have focused on the involvement of telomerase activation in the immortalization of cancer cells and cellular rejuvenation. However, some studies demonstrated that the results do not meet expectations for telomerase action in telomere maintenance. Recent results give reason to think that major telomerase components—the reverse transcriptase protein subunit and telomerase RNA—may participate in many cellular processes, including the regulation of apoptosis and autophagy, cell survival, pro-proliferative effects, regulation of gene expression, and protection against oxidative stress. However, the difficulties faced by scientist when researching telomerase component functions often reduce confidence in the minor effects observed in experiments. In this review, we focus on the analysis of the functions of telomerase components (paying more attention to the telomerase RNA component), both as a complex and as independent components, providing effects that are not associated with telomerase activity and telomere length maintenance. Despite the fact that the data on alternative roles of telomerase components look illusory, it would be wrong to completely reject the possibility of their involvement in other biological processes excluded from research/discussion. Investigations to improve the understanding of every aspect of the functioning of telomerase components will provide the basis for a more precise development of approaches to regulate cellular homeostasis, which is important for carcinogenesis and aging.

## 1. Introduction

Special structures known as telomeres are located at the ends of linear chromosomes and protect them from shortening and fusion [1]. Telomeres shorten due to the end replication problem [2] and nucleolytic degradation [3]. Telomerase reverse transcriptase (TERT) uses a telomerase RNA (TERC—telomerase RNA component) as a component of the telomerase complex and a template to add telomeric repeats to the 3′-end of chromosomal DNA [4,5,6,7]. Telomerase RNA provides the structural scaffold for telomerase complex assembly [8]. Telomere length defines the cell’s lifespan, and critically short telomeres promote the activation of DNA damage signaling, resulting in cell death [9,10,11]. Elongation of telomeres by telomerase provides unlimited proliferative potential for the cell [12,13]. Telomerase activity is inherent to stem, germ, and the majority of cancer cells. Traditionally, telomerase investigations have been associated with cancer and aging. However, the expression of *hTERT* has been demonstrated for cells with active telomerase while *hTERC* gene expression is not correlated with telomerase activity [14]. hTERC has been detected in the majority of somatic cells where it is expressed constitutively. Mutations in *hTERT* and *hTERC* genes are involved in the development of diseases linked to dysfunctional telomeres, such as aplastic anemia, the autosomal dominant form of dyskeratosis congenita [15,16,17], and idiopathic pulmonary fibrosis [18]. This review is focused on different aspects of telomerase ribonucleoprotein (RNP) components functioning in both the complex and separately, with special attention paid to the RNA component.

## 2. Structure of Human Telomerase RNP

**Figure 1:**
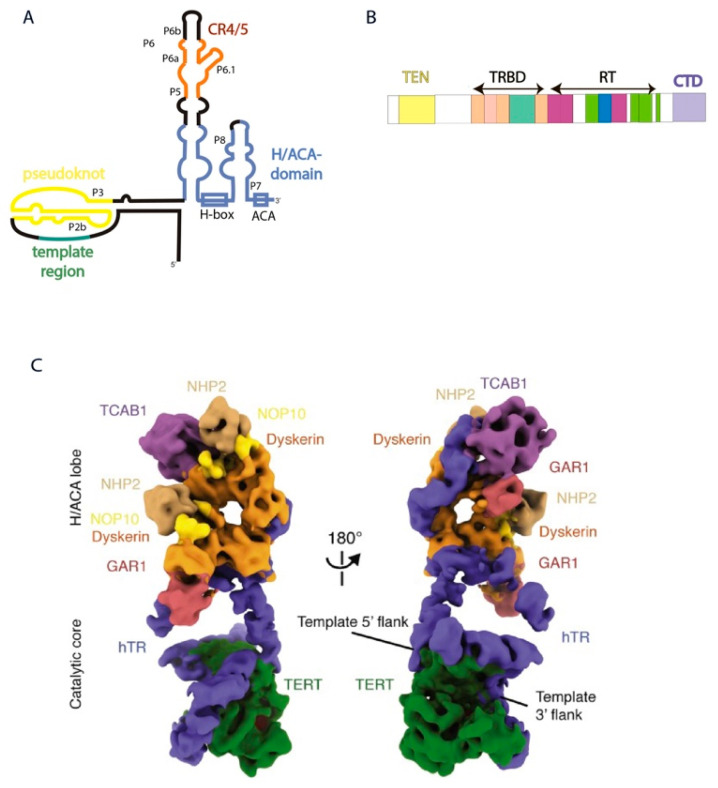
Human telomerase structure. (**A**) Secondary structure of hTERC. (**B**) Domain architecture of hTERT. (**C**) Cryo-EM structure of the human telomerase holoenzyme in two views [26] (Adapted with permission from Elsevier). Subunits are colored as labeled.

Catalytically-active RNP purified from cells as a complex contains TERT, TERC, dyskerin (DKC), NOP10, NHP2, GAR1, and TCAB1 [8]. DKC1, NOP10, NHP2, and GAR1 are shared by telomerase and the small nucleolar (sno) RNPs, and TCAB1 is a component of small Cajal body (sca) RNPs. snoRNP and scaRNP catalyze ribosomal and spliceosomal RNA modifications, respectively. However, telomerase activity may be reconstituted in vitro by the complex of two major components: TERT and TERC. hTERC contains 451 nucleotides (nts) that form several structural domains (Figure 1A). However, only two domains are necessary for telomerase activity to occur. Pseudoknot containing the template region (t/PK) and H/ACA-domain of hTERC expressed separately are able to reconstruct the minimally catalytically-active telomerase enzyme that is capable of synthesizing the telomere repeats (Figure 1A). t/PK possesses the template for telomere synthesis and is involved in association with hTERT. Interestingly, the presence of a phylogenetically-conserved hairpin in equilibrium with the pseudoknot in the telomerase pseudoknot domain, which is stabilized by a unique uridine helix, has been demonstrated. A functionally important interconversion between the hairpin and pseudoknot conformations was proposed [19]. The H/ACA domain of hTERC shared with snoRNAs is important for hTERC biogenesis as well as for telomerase activity. It contains CR4/5 (conservative regions 4 and 5) which are responsible for the binding of hTERT and CAB-box, providing the signal to localization in Cajal bodies similar to other known scaRNAs specific to this intranuclear compartment [20,21].

TERT contains four domains (Figure 1B): TEN-domain (telomerase N-end domain), TRBD (telomerase RNA-binding domain), RT (reverse transcriptase domain), and CTD (C-end domain). hTERC binding to H/ACA-proteins is the first step of human telomerase RNP assembly. Two hetero-tetramers of H/ACA proteins interact with the H/ACA-domain of hTERC in different positions. One molecule of DKC1 attracts the H/ACA-complex to attach to the P4 stem of hTERC. The other dyskerin molecule strongly interacts with the P7 stem of hTERC, attracting NOP10, NHP2, and TCAB1 to the P8 stem (Figure 1) [8].

The interaction of TCAB1 is absolutely necessary for the assembly of the catalytically-active telomerase complex. Binding of TCAB1 with hTERC promotes the formation of the tertiary structure of the CR4/5-domain, which is preferential for the association with hTERT. The absence of TCAB1 or mutations of *TCAB1* or in the CR4/5-domain of hTERC disturb the interaction of hTERT with hTERC and lead to a decrease in the telomerase activity and shortening of telomeres in human embryonic stem cells [22].

The TRBD (telomerase RNA-binding domain)-domain of hTERT interacts with the t/PK and CR4/5-domains of hTERC (Figure 1A,B) [23,24]. The P6.1 stemloop of hTERC is critical for the formation of active enzyme. Mutations in P6.1 that disturb its secondary structure impair the interaction between hTERT and hTERC and decrease telomerase activity, while compensatory mutations that restore the secondary structure of this element recover the activity of telomerase [20,25]. To form the catalytic center of the human telomerase holoenzyme, the pseudoknot and CR4/5 domain of hTERC should wrap around hTERT (Figure 1C). PK adopts an arch-shaped structure where a triplex structure formed from the P2b and P3 stems brings the TRBD and CTD-domains of hTERT together. The template region of hTERC occurs nearby to the TEN-domain of hTERT that stabilizes the 3′-part of the RNA-DNA duplex formed by telomere and hTERC [8]. 

Stems P5, P6 and P6.1 form the three-way junction (TWJ) element involved in the interaction with hTERT. P6a stem located along TRBD of hTERT, while P6.1 exudes from the TWJ almost perpendicularly to P6a located between the TRBD and CTD domains of hTERT [8]. The CTD domain localizes opposite the P6.1 stemloop, stabilizing this structure that is important for human telomerase activity (Figure 1) [26].

## 3. Telomerase Action at Telomeres

Telomere lengthening due to the reverse transcriptase activity of TERT is recognized as a major intracellular function of the telomerase complex. TERT adds telomere repeats using its own telomerase RNA as a template and 3′-OH group of the telomere as a primer. The TEN domain of hTERT stabilizes the RNA–DNA duplex formed by the template region of telomerase RNA and telomeric DNA near to the catalytic center of the enzyme.

**Figure 2:**
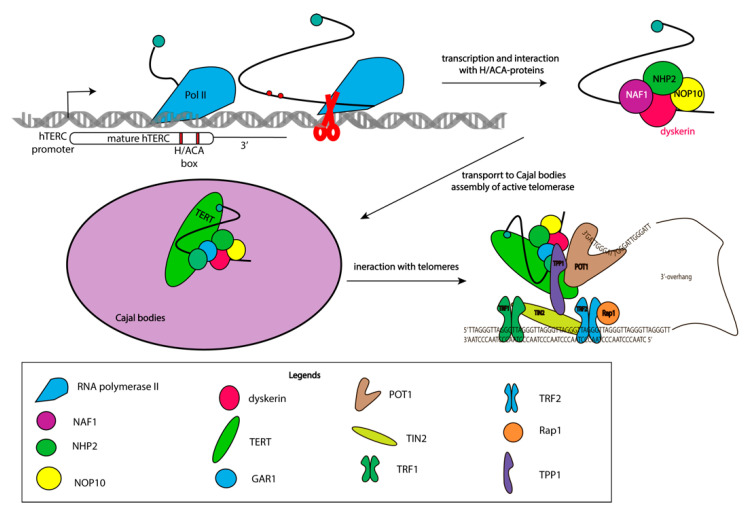
Multistage process of assembly of the active telomerase complex. Scheme illustrating different stages of telomerase complex assembly and its binding to telomeres.

Telomerase RNP intracellular trafficking between Cajal bodies, the nucleolar, and the nucleoplasm occurs during biogenesis. DKC1, NOP10, NHP2, and NAF1 interact with hTERC co-transcriptionally, and assembled complexes rapidly transfer to Cajal bodies where trimethylation of the 5′-cap of hTERC occurs [27]. DHX36 RNA-helicase unfolds G-quadruplexes formed at the 5′-end of hTERC. GAR1 replaces NAF1, and the complex of hTERC with H/ACA proteins interacts with hTERT and TCAB1 to form a catalytically active enzyme [28]. Approximately 100 molecules of active telomerase RNP are formed in every cell cycle [29,30], which is not enough to set up experiments under conditions of an endogenous level of expression. Indeed, the formation of active telomerase RNP in nucleolar was demonstrated with the exogenous expression of hTERT and a decreased level of H/ACA proteins. In absence of TCAB1 the assembly of active telomerase RNP occurs in the nucleus without the activation of hTERT expression [31]. Thus, active telomerase complexes may be formed in Cajal bodies or nucleoplasm (Figure 2).

TCAB1 interacts with active and inactive telomerase complexes and promotes their trafficking from the nucleolar to the nucleoplasm and Cajal bodies in the G1 phase of the cell cycle [29,32,33]. Coilin dysfunction resulting in the absence of Cajal bodies does not influence the assembly of active telomerase RNP, and the telomere length in cancer and embryonic stem cells suggest the effective interaction of telomerase with telomeres in the nucleoplasm [31,34]. Co-localization of telomerase with telomeres and Cajal bodies clearly demonstrates that the interaction of telomerase with telomeres occurs in the S-phase of the cell cycle in the nucleoplasm, but not in Cajal bodies [35]. 

The interaction of telomerase with telomeres is promoted by the TPP1 protein in vertebrates. The structural OB-fold domain of TPP1 binds to the TEN domain of telomerase reverse transcriptase to position RNP at the single strand region of telomeres, facilitating the interaction of the template region of telomerase RNA with the end of the chromosome [36]. 

**Figure 3:**
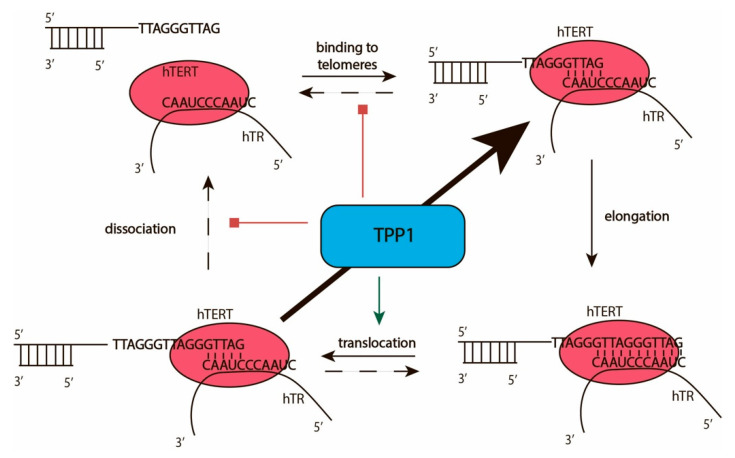
Catalytic cycle of telomerase. Scheme illustrating the telomerase active cycle and its modulation by TPP1.

The interaction of telomerase with telomeres facilitates the addition of telomeric repeats to the 3′-end of the telomere overhang. Telomerase catalyzes the addition of deoxynucleotide triphosphate (dNTPs) to the 3′-OH group of 2′-deoxyribose of the last telomeric nucleotide by the formation of phosphodiester bonds according to the template region of TERC. The efficiency of telomerase action may be characterized by two types of processivity: types I and II (Figure 3). Processivity of the type I provides information concerning the number of nucleotides added to the end of telomerase without telomerase–telomere complex dissociation, which may occur after the addition of a single nucleotide [37]. The number of telomeric repeats added to individual telomeres without dissociation of telomerase characterizes the type II processivity of the enzyme (Figure 3). The addition of each repeat followed by melting of the DNA–RNA duplex and translocation of telomerase in a way that transfers the template region of telomerase RNA to stimulate hybridization at the 3′-end of the telomere and correct arrangement of the heteroduplex in the active center of telomerase. The maximal RNA–DNA-duplex may be formed by the complementary interaction of 11 nucleotides of telomerase RNA and telomeric DNA. The optimal DNA–RNA duplex length is 5–6 nucleotides [38]. The additional nucleotides stabilize the structure and prevent the effective melting and translocation of telomerase. Recent structural data indicates that the TEN domain of telomerase reverse transcriptase limits the length of the product-template heteroduplex [8,39]. The TEN domain binds to the DNA/RNA duplex at the bifurcation point and to some regions of TERC outside the DNA/RNA duplex, as has been demonstrated for *Hansenula polymorpha* [39] and confirmed later for human telomerase [8]. The telomeric protein TPP1 stimulates translocation and reduces the dissociation efficiency of the complex of telomerase and telomere, increasing the number of telomeric repeats added during one act of telomerase action without dissociation from the telomere [40].

Telomerase was first recognized as a telomere-maintaining enzyme due to its reverse transcriptase activity. However, extensive investigations of telomerase have provided data detailing possible additional functions of components of the telomerase holoenzyme that are not associated with telomere lengthening. Both major components of the telomerase complex may be involved not only in oncogenesis but in different physiological intracellular mechanisms, such as tissue homeostasis, gene expression, and the stress response. The alternative functions of TERT were reviewed in detail recently [41,42], so in this review, we concentrate on the non-telomeric action of telomerase RNA.

## 4. Effects of the Expression of *TERC* at the Early Stages of Tumorigenesis Are Not Associated with Telomerase Activation

The first evidence that telomerase RNA can function independently of telomerase was observed in investigations of the expression of telomerase components and the activation of telomerase following tumorigenesis in mice. The activation of *mTerc* expression was demonstrated in two different transgenic mice models of multi-stage tumorigenesis: K14-HPV16 and RIP-TAg2 [43]. The K14-HPV16 mice expressed the human papillomavirus type 16 region in basal keratinocytes. This model allows the multistage induction of squamous cell carcinomas of the epidermis to be controlled. RIP1-TAg2 mice develop islet cell carcinomas by a multistage process controlled by the expression of the SV40 T antigen (TAg) oncogene in pancreatic β-cells. The analysis of telomerase activity and *mTerc* expression revealed upregulation of the telomerase RNA component at very early stages of tumorigenesis in both used models, whereas telomerase activity was detected in end-stage tumors [43]. The possible alternative function of TERC as a response to the initiation of continuous cell proliferation may explain the upregulation of *TERC* expression at the early stages of tumorigenesis independent of telomerase activation. However, we cannot exclude that early overexpression of *hTERC* is necessary in order to obtain a sufficient concentration of this molecule ready for effective telomerase complex assembly at later stages when the overexpression of hTERT will occur.

Additional evidence of the noncanonical role of the telomerase RNA component in tumorigenesis was observed in K5-*Tert* mice overexpressing *TERT* in basal keratinocytes from stratified epithelia [44]. K5-*Tert* mice demonstrated increased susceptibility to skin tumorigenesis and an increased rate of wound healing than wild-type mice without the telomere length being affected. Model mice were generated in a *Terc^-/-^* genetic background to reveal the role of the telomerase complex in tumorigenesis. An impairment of tumorigenesis in K5-*Tert/Terc^-/-^* mice in comparison to control *Terc^-/-^* mice was observed. The authors concluded that *Tert* overexpression inhibits tumorigenesis in the absence of *Terc,* and this effect is independent of telomerase activity and telomere length. A delayed rate of wound healing in K5-*Tert/Terc^-/-^* mice in comparison to control *Terc^-/-^* mice indicates that *Terc* is necessary for a higher wound healing rate, and this effect is independent of telomerase activity and telomere length [44]. It is interesting that the overexpression of *Tert* does not promote increased wound healing and increased tumorigenesis. Expression of *Terc* might be necessary at the early stages of tumorigenesis in mammals, which correlates with the amplification of genome locus 3q26 containing *Terc* in human tumors [45]. However, the data obtained in this research must be interpreted very carefully, and it is very difficult to separate effects provoked by the telomerase components or telomere length.

The influence of telomerase RNA on the regulation of the cell growth rate was uncovered in experiments involving the inhibition of *hTERC* expression by RNA interference [46]. The distinct effects of inhibition telomerase activity and telomere shortening were observed after long-term treatment. The depletion of the telomerase RNA component reduced the cell proliferation rate quickly, without having a significant influence on the level of telomerase activity. At this stage, *hTERC* knockdown does not cause telomere uncapping or a DNA damage response, but it induces changes in global gene expression [46]. Rapid downregulation of genes involved in cell cycle progression, such Cyclin G2 and Cdc27, was observed. Interestingly, the knockdown of *TERC* also rapidly lowered the expression of specific genes coding for proteins important for tumor growth, angiogenesis, and metastasis, such as integrin αV and Met oncogene protein [26].

Interestingly, two copies of the telomerase RNA component are encoded in Marek’s disease virus (MDV) [47]. MDV is a lymphotropic alphaherpesvirus that causes Marek’s disease (MD) in chickens. This condition is associated with neurological disorder, immune suppression and, primarily, malignant T cell lymphomas [48]. Viral telomerase RNA (vTERC), which shares 88% sequence identity with chicken telomerase RNA (chTERC), is expressed during both lytic and latent MDV infection. vTERC can reconstitute telomerase activity with chicken vTERT in vitro [29]. However, it was demonstrated that the tumorigenesis effect in virus-induced lymphomas of vTERC occurs independently of telomerase activity. The mutant form of vTERC that does not interact with chTERT and is incapable of activating telomerase activity demonstrated the same tumorigenesis effect as wild type vTERC [49]. Expression of *vTERC* in chicken fibroblasts (DF-1) led to a two-times increase in integrin αV expression [48]. RNA pull-down assay can identify RPL22, a ribosomal protein that is involved in T-cell development and virus-induced transformation, as a protein interacting with vTERC and chTERC [49]. However, the interaction of RPL22 with vTERC was found to be two times stronger in comparison with chTERC. The expression of *vTERC* led to the re-localization of RPL22 from the nucleolus to the nucleoplasm, which is similar to the effect of EBER-1, a small type of RNA that contributes to tumor formation upon Epstein–Barr virus infection. The re-localization of RPL22 due to its interaction with EBER-1 is associated with an enhanced proliferative potential of cells [50,51]. Notably, the interaction of hTERC with RPL22 has been demonstrated in vivo [52]. 

These observations, therefore, suggest that telomerase activation promotes tumorigenesis and immortalization at different levels of regulation, and telomerase components may have additional contributions, independent of their essential functions in telomere elongation. However, the fact that the expression levels of telomerase components and the rate of telomerase activity influence the telomere length, which is strongly associated with cell cycle progression and cell proliferation, make it impossible to separate effects initiated by the telomere length or noncanonical functions of the telomerase component. This question should be investigated carefully to allow to confidently conclude that telomerase RNA has some functions in the regulation of the proliferation rate as an individual molecule.

## 5. TERC in the Regulation of Gene Expression

**Figure 4:**
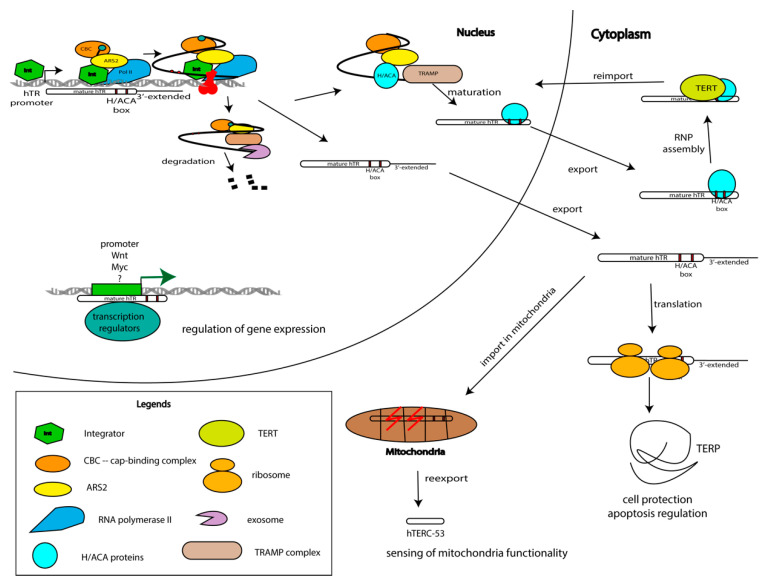
Model of hTERC biogenesis, depicting the competition between processing and degradation, and trafficking through cellular organelles with the distribution of functions. Telomerase RNA is synthesized as a long precursor that may be degraded, processed to the mature form following association with hTERT or it may work outside of the telomerase complex. TERC is involved in gene expression regulation due to its interaction with promoter regions of genes involved in the regulation of proliferation and cell cycle progression. TERC is transported from the nucleus to the cytoplasm where it may associate with TERT and be reimported to the nucleus to elongate the telomeric repeats. On the other hand, TERC is imported to the mitochondria where it is processed and reexported to the cytoplasm. The precursor of TERC is translated and the obtained TERP protein protects cells under stressful conditions.

The potential mechanism of TERC’s participation in the regulation of global genome expression may be derived from experimental genome mapping of long noncoding RNA occupancy. The improved method, termed CHIRP (Chromatin Isolation by RNA Purification), allows the discovery of RNA-bound DNA and proteins. Significant enrichment of telomerase RNA at telomeric regions has been demonstrated, as was expected. Surprisingly, numerous specific binding sites for hTERC have been detected throughout the genome. A total of 2198 hTERC binding sites have been identified in the genome with a covering pattern very similar to that of the transcription factors. It was observed that TERC preferably binds to the cytosine-rich motif 5′-GGCCACCACCCC-3′, which is complementary to the sequence 5′-GGGGUGGUGGCC-3′ at nucleotides 25–36 of hTERC. It was shown that hTERC binding sites are located at the multiple *Wnt* genes and a series of *Myc* genes [53] (Figure 4), which correlates with the previously documented binding sites of TERT. The activation of *Wnt* reporters by TERT in *TERC^-/-^* mouse embryonic fibroblasts was demonstrated previously [54]. Thus, one can speculate that the binding of TERC to specific sites on DNA may have a direct effect on transcription or an indirect effect by attracting TERT via the telomerase complex. Independent TERT binding to promoter regions cannot be excluded. 

Evidence shows that the binding of TERC to DNA can directly influence the transcription pattern was obtained in recent work on the identification of the upregulation of genes related to the immune system in telomerase-negative U2OS cells that ectopically express *hTERC* [55]. The stimulation of TERC-U2OS with TNF-α led to the upregulation of cytokine expression and increased its secretion in culture medium independently of telomerase activity. The depletion of hTERC by RNA interference resulted in decreases in both the expression and secretion levels of cytokines. The activation of the NF-κB pathway in *TERC*-expressing cells in comparison with TERC-negative cells was shown. Analysis of binding sites of TERC on chromosomes demonstrated that the majority of them were located within ±1000 bp of transcriptional start sites. For several genes related to the NF-κB pathway—*LIN37*, *TPRG1L*, *TYROBP*, and *USP16*—the direct interactions with hTERC were demonstrated in vitro. Further in vivo experiments revealed upregulation of these genes as a response to *TERC* expression. Interestingly, the increased expression level of *hTERC* was determined in CD14+ cells from type II diabetes and multiple sclerosis patients displaying increased chronic inflammation [55]. The expression levels of *TPGRL1* and *TYROBP* and *IL-8* and *TNF-α* increased for diabetic patients, whereas *TYROBP* and *USP16* and *IL-6*, *IL-8*, *CSF2,* and *TNF-α* increased for multiple sclerosis patients. The fact that U2OS cells are telomerase-negative allows us to affirm that the regulation of NF-κB-pathway occurs due to direct action of TERC and is independent of the telomerase complex and telomere length.

TERC occupied sites near *CSF1* and *CSF2* genes encode macrophage colony-stimulating factor (MCSF) and granulocyte-macrophage colony-stimulating factor (GMCSF), as was revealed by CHIRP approach [53]. Decreased expression was demonstrated for the *CSF1* and *CSF3* genes in TERC-deficient larvae of zebrafish [56], while the overexpression of *TERC* increased the levels of both transcripts. The expression of the transcriptional factors spi1 and gata1a, which are essential for the differentiation of hematopoietic stem cells (HSCs), was affected in zebrafish embryos when TERC was depleted. *GCSF* overexpression has been shown to restore the expression level of the major myeloid transcription factor *spi1* and does not affect TERC levels. This indicates that TERC participates in the regulation of the expression of *gcsf* and *mcsf* genes, which maintain a critical balance between the major myeloid (spi1) and erythroid (gata1) transcriptional factors [56]. Finally, the depletion of TERC in zebrafish embryos results in strong neutropenia and monocytopenia that is characterized by a decreased number of fully functional neutrophils and macrophages. This effect is fully rescued by the overexpression of TERC. The effect of TERC in developmental myelopoiesis is independent of telomerase activity and telomere length. 

Thus, the interaction of TERC with the promoter areas of specific genes (Figure 4) provides a basis for the participation of TERC in transcription regulation, at least in genes of the immune system and myelopoiesis. 

## 6. TERC and Cell Protective Mechanisms

TERC can affect cellular processes, not only by the modulation of gene expression but by its influence on cellular signaling systems.

Multiple studies have provided evidence concerning the implications of TERC in cell protective mechanisms. Protein kinase ATR (ATM (ataxia telangiectasia mutated) and Rad3-related) participates in the modulation of cellular responses to DNA damage. ATR is activated when single-stranded DNA ends emerge upon the formation of DNA adducts, during the processing of double-strand breaks (DSBs), or during termination of the replicative fork [57,58]. Suppression of ATR kinase activity is observed under conditions of increased expression of *hTERC* [59]. Reduction of the hTERC level stimulates ATR activity. These processes are independent from the level of telomerase activity and the telomere length. A reduction of *hTERC* expression in cells results in an increase in the amount of p53, the tumor suppressor and major contributor to the signaling pathway under conditions of oncogenic stress. Meanwhile, the cell content of the protein CHK1, the cell cycle regulator, increases. p53 and CHK1 are the major substrates of ATR kinase. hTERC inhibits ATR kinase and disrupts the regulation of cell cycle checkpoints following DNA damage in vivo [59].

The activation of DNA-dependent protein kinase (DNA-PK), related to the same PIKK (phosphatidylinositol 3-kinase-related kinases) family as ATR, by hTERC [60] was described several years later. It was demonstrated that DNA-PK phosphorylates the hnRNP A1 protein in the presence of hTERC in vitro and in vivo. hnRNP A1 is involved in the regulation of alternative splicing and was identified as a telomere-associated protein and suggested to be a regulator of the recruitment of telomerase to chromosome ends. Unfortunately, the activation of DNA-PK by hTERC has only been studied in terms of its relationship with hnRNP A1. However, it was shown that the activation of DNA-PK occurs in a hTERC-dependent manner in vitro and in vivo. The exogenous expression of *hTERC* in VA13 cells lacking telomerase RNA was shown to increase the level of phosphorylated hnRNP A1 [60], which was reduced by treatment with the inhibitor of DNA-PK. Data obtained in this study allowed the proposal that hTERC is involved in the regulation of the DNA damage response by the activation of DNA-PK, which is required for the repair of double-strand breaks via a nonhomologous end-joining pathway [61,62]. Although evidence that telomerase activity and the telomere length do not influence the effect of the hTERC level on the regulation of the DNA damage response has been provided, it is very difficult to exclude the idea that telomerase RNA may be involved in the structural organization of telomeres. Indeed, DNA-PKs are known to associate with telomeres and regulate the cellular response to telomere dysfunction through the p53 pathway. Ectopically-expressed telomerase RNA in telomerase-negative cells may interact with telomeres and disturb their structure, which will influence the DNA damage response. 

Surprisingly, mitochondrial trafficking of TERC was recently demonstrated (Figure 4) [63]. The mitochondria import signal—the first 52 nucleotides—was identified in hTERC. The processing of hTERC to the shorter form hTERC-53 corresponding to the 52–248 nt of hTERC occurs in mitochondria followed by export back to the cytoplasm [63]. hTERC-53 accumulates in the cytoplasm when the membrane potential of mitochondria is impaired (Figure 4). More severe damage to mitochondrial functions could lead to dramatic changes in the cytosolic hTERC-53 level because of mitophagy or apoptosis [63]. The cellular and physiological functions of hTERC-53 and the mitochondrial trafficking and processing of hTERC should be investigated more carefully. 

The presence of an additional alternative *TERC* gene in mice was revealed by searching the mouse genome by BLAT [64]. Alternative TERC (alTERC) showed an 87.9% similarity to mTERC. The differences were caused by the deletion of 18 nt in the CR4 region of alTERC. The expression of alTERC in mouse brain was determined by reverse transcription followed by PCR. AlTERC is associated with TERT and supports telomerase activity. Overexpression of *mTERC* and *alTERC* protects motor neuron cells from oxidative stress [64]. The survival of cells expressing *alTERC* increased in comparison with the survival rate of cells with an increased level of canonical *mTERC*. The observed protective effect occurred independently of TERT expression or telomerase activity.

The increased expression of telomerase components and telomerase activity are associated with the stimulation of T-cell proliferation [65,66,67,68]. Interestingly, an increased level of *hTERC* expression is important for short-term CD4 T-cell survival, while telomerase activity is needed for long-term survival [69]. The cell protective effect of telomerase RNA overexpression independent of telomerase activity was demonstrated under dexamethasone treatment. Overexpression of wild type hTERC results in the stimulation of telomerase activity and does not have an antiapoptotic effect. In addition, the expression of chimeric RNA hTERC-U64, which contains the 5′-half of hTERC (pseudoknot and template region), occurs, but the 3′ half (CR4/CR5 and box H/ACA regions) is replaced by the H/ACA domain of the similarly sized U64 small nucleolar (sno)RNA [69]. Chimeric RNA cannot associate with hTERT and activate telomerase. Overexpression of mutant forms of telomerase RNA Δ96–97 and G305A, but not wild type or chimeric TERC, protects cells against dexamethasone-induced apoptosis. The hTERC mutation Δ96–97 shifts the structural equilibrium from a pseudoknot structure to a hairpin, which abolishes telomerase activity [19]. The point mutation G305A in hTERC reduces the binding to hTERT by up to 80%. Moreover, wild-type hTERC, as well as both mutant forms of hTERC, protect CD4 T-cells against apoptosis when the hTERT level is reduced. As expected, the reduction of hTERC level was shown to induce apoptosis in CD4 T-cells. The genome-wide microarray analysis did not reveal any transcriptional changes in genes involved in apoptosis in cells with a reduced level of hTERC. The overexpression of wild type *hTERT* but not of the catalytically inactive isoform led to apoptosis induction and the simultaneous expression of catalytically inactive *hTERC* mutants restored the protective potential [69]. Altogether, these data provide evidence that the telomerase RNA component may protect cells against apoptosis through its alternative function.

The data described above represent a set of evidence of the involvement of hTERC in various cellular processes in the nucleus, cytoplasm, and even the mitochondria, independently of telomerase and transcription activation, but do not explain the molecular mechanisms of the telomerase RNA functions outside the telomerase complex (Figure 4). 

The set of hTERC transcripts with heterogenous 3′-ends appears in the process of transcription of the *hTERC* gene [70]. The position of the point of transcription termination is undetermined and the length of primary transcript of hTERC is unknown at this moment. However, the application of the deep-sequencing has revealed the major fraction of hTERC transcripts corresponding to the mature form (451 nt). Additionally, a fraction of transcripts lengthened for 5–6 nts are encoded in the genome, followed by the oligo(A)-sequence [71]. The stable intermediates with diverse lengths should appear during the rapid processing of the elongated (up to 1000 nts) primary transcript of hTERC [70]. 

Multisubunit’s complex Integrator regulates the termination of *hTERC* transcription in a promoter-dependent manner. Depletion of the Integrator results in the accumulation of the precursor elongated by up to 571 nucleotides [72]. Different extended forms of the hTERC primary transcript have been detected: some of them contain only few additional nucleotides, whereas some of them exceed 1500 nt in length [70]. 

The main function of telomerase is in the nucleus, and the majority of hTERC is located in the nucleus. However, under certain conditions, hTERC can be found in the cytoplasm. It was shown that the depletion of DKC1 leads to the appearance of hTERC in the cytoplasm [73]. The loss and mutations of TGS1 promote the accumulation of hTERC in the cytoplasmic fraction [74]. Moreover, accurate analysis of wild type HEK293T cells revealed that around 20% of hTERC is localized in the cytoplasm [75]. 

The transcript of hTERC contains an open reading frame (ORF) that starts at position 176 nt and codes for the protein named hTERP. hTERP contains 121 amino acid residues and is encoded in a premature transcript. It should be mentioned that hTERC was shown to be associated with the ribosomes according to ribosome profiling data [76,77] and it is present in the polysome fraction [78]. The localization of the AUG codon in the pseudoknot domain should impede its translation; however, the appearance of an alternative hairpin structure [19] allows start codon recognition and translation of the protein hTERP. 

The existence of hTERP has been confirmed by several experimental approaches, including mass spectrometry and immunodetection [75]. It was revealed that overexpression of wild-type *hTERC*, but not of the mutant which is incapable of directing hTERP synthesis (mutation in start-codon), protects HEK293T cells from doxorubicin-induced apoptosis. Moreover, mutations at the N-terminus of hTERP and a reduced level of hTERP affect autophagosome formation [75]. hTERP may be involved in the protection of cells from stress and helps cells to survive and adapt to unfavorable conditions (Figure 4). 

The coding potential of hTERC explains the constitutive expression of *hTERC* independently from telomerase activity. Upregulation of the expression of *TERC* in the early stages of tumorigenesis may be necessary to increase the level of TERP protein that protects cells from apoptosis and promotes survival in the period when the cell should adapt to the new metabolism. Mutations in TERC promote severe phenotypes associated with telomeropathies, such as dyskeratosis congenita [17]. Mutant forms of TERC, which are not able to associate with TERT but save open reading frames protect cells from apoptosis [69]. In this case, the accumulation of unbound hTERC may promote translation, and an increased level of hTERP protects cells from apoptosis (Figure 4).

Recent work revealed that hTERC produces small RNA named terc-sRNA at positions 425–447 nt. The interaction of terc-sRNA with AGO2 affects telomerase complex formation and telomere lengthening. The expression level of AGO2 correlates with telomerase activity. Surprisingly, authors demonstrated that the overexpression of terc-sRNA in cells knocked out by AGO2 stimulates telomerase activity. Telomerase RNA itself has been identified as target RNA for terc-sRNA, so it may be involved not only in the regulation of telomerase assembly but it may also influence translation and the level of hTERP protein expression in cells [79]. 

Thus, the majority of cell protective functions of telomerase RNA, excluding transcription regulation, may be explained by the appearance of the protein hTERP, which is encoded in this RNA. Indeed, the protective effect of the hTERP protein encoded in hTERC was demonstrated in cells under apoptosis-inducing conditions. The hTERP protein is involved in autophagosome formation. The coding capacity of hTERC may explain the protective effect under apoptosis-induction treatment of immune cells of mutant hTERC, which is incapable of forming a complex with hTERT. 

## 7. Conclusions and Perspectives

The major function of telomerase complex components is related to the maintenance of the telomere length. The majority of investigations on telomerase have been concentrated in the field of aging and cancer biology for a long period. Investigations aiming to understand the roles of TERT or TERC in telomerase activation and disease development have provided data showing the possible functional role of telomerase components outside of telomerase. The overexpression of *TERC* and *TERT,* which is independent of telomerase activation, may promote the increased survival of transformed cells due to the protective function of TERC itself or may activate the expression of components of regulatory cascades involved in proliferation and cell protection. 

The data concerning the alternative function of telomerase components in cell protective mechanisms may be considered to show a new level of regulation related to tumorigenesis, telomere syndromes, and cell survival. However, at this moment, we have fragmented data concerning the action of telomerase components outside of the telomerase complex, and it is very difficult to separate their functions as individual components or as compounds of the telomerase machine. More precise investigations of telomerase components’ functions and the mechanisms of expression regulation under different states of cellular homeostasis will enhance the development of approaches for the modulation of carcinogenesis and aging.
